# Winner of the Ronald Melzack–*Canadian Journal of Pain* 2022 Paper of the Year Award/Récipiendaire du Prix Ronald Melzack Pour L’Annee 2022 des Articles Parus Dans la *Revue Canadienne de la Douleur*

**DOI:** 10.1080/24740527.2023.2254576

**Published:** 2023-09-05

**Authors:** Jo Nijs, Joel Katz

**Affiliations:** Pain in Motion Research Group (Pain), Department of Physiotherapy, Human Physiology and Anatomy, Faculty of Physical Education & Physiotherapy, Vrije Universiteit, Brussels, Belgium; Chronic Pain Rehabilitation, Department of Physical Medicine and Physiotherapy, University Hospital, Brussels, Belgium; Department of Health and Rehabilitation, Unit of Physiotherapy, Institute of Neuroscience and Physiology, Sahlgrenska Academy, University of Gothenburg, Gothenburg,Sweden; Psychology, York University, Toronto, Ontario, Canada

Each year, the Canadian Pain Society (CPS) and its official journal, the *Canadian Journal of Pain*, present an award in honor and memory of the late Ronald Melzack, a Canadian psychologist and the world’s preeminent pioneer in pain theory, research, and management. This year, the Ronald Melzack–*Canadian Journal of Pain* Paper of the Year Award for articles published in 2022 was won by Nathan Augeard, Dr. Timothy Wideman, and colleagues for their article titled “Development of a National Pain Management Competency Profile to Guide Entry-Level Physiotherapy Education in Canada.”^1^ The winning paper was selected by the editor-in-chief, Joel Katz, from a short-list of six original articles published in the journal in 2022 based on rankings submitted by 23 journal editorial board members who considered originality, novelty, quality, and potential impact when submitting their votes. The Augeard et al. article was the clear winner based on the number of editorial board members who ranked it as their number one pick.

The 2022 Paper of the Year Award was presented by Joel Katz to co-author and national pain community leader, spokesperson, and advocate Lynn Cooper, who accepted it on behalf of the authors at the 43rd Annual Scientific Meeting of the Canadian Pain Society gala dinner awards ceremony in Banff, Alberta. In their prepared remarks, the authors stated, “This CIHR [Canadian Institutes of Health Research]-funded study reflects the collaborative efforts of a diverse group of 28 co-authors, including researchers, physiotherapy educators and people living with pain. The paper aims to create a new national standard for how physiotherapists are trained to manage pain across Canada.” Indeed, the authors conducted a very rigorous study using a modified Delphi approach with in-depth input from a comprehensive team of stakeholders. This resulted in a consensus-based list of competences required by physiotherapists to manage pain upon entry to practice specific to the Canadian physiotherapy context. This competence profile is very important and highly relevant, probably beyond the Canadian physiotherapy context. Once implemented, it holds potential to improve physiotherapists’ pain management practices worldwide and, consequently, to improve quality of life and reduce pain substantially in a large number of patients with (persistent) pain.

The creation of the competence profile is the first and key step to take physiotherapy management of pain to the next level. On the other hand, some may say that these competences are too generic, because they may also apply to managing other conditions (e.g., managing chronic fatigue in cancer survivors or patients with arthritis or physiotherapy for stroke patients). Specific competences for managing (persistent) pain such as assessment and treatment of relevant lifestyle factors^2,3^ would have been useful, including a position statement about physiotherapists’ role in dealing with stress intolerance,^4^ poor sleep,^5^ and dietary habits^6^ (beyond the mentioned physical activity). This issue is briefly acknowledged by the authors in the discussion section,^1^ but a focus on competences (referred to the authors as “our focus on the *does* level” p. 9) in no way prevents addressing these factors in the future. The role of physiotherapists in providing such lifestyle interventions remains to be established in light of the growing evidence supporting the integration of sleep management^7^ and dietary interventions^8^ in the multimodal management of chronic pain.

In undergraduate programs, physiotherapists typically receive little education and training regarding their role in nutrition care for pain management and therefore may pass on opportunities to counsel their patients on the connection between nutrition and pain.^9^ Likewise, sleep disorders and their management, although highly prevalent in physiotherapy practices (e.g., in patients with spinal pain^10^ and cancer survivors^11^), are often overlooked in physiotherapy curricula, yet at the very least physiotherapists are very well suited to deliver sleep education and sleep hygiene in a stepped care approach,^12^ with subsequently only the nonresponders requiring more comprehensive sleep treatment (i.e., cognitive–behavioral therapy for insomnia) by a sleep expert.

As explained in the paper, some stakeholders wanted more detailed content (e.g., best practice recommendations) to inform the practical applications of the competency profile,^1^ which will be key to develop as soon as possible, because research has informed us that physiotherapists tend to adhere poorly to best practice guidelines for the management of persistent pain.^13,14^ Several studies have shown that physiotherapists’ attitudes and beliefs determine guideline adherence (e.g., for the management of osteoarthritis and low back pain^13,15–17^); for example, physiotherapists who hold biomedical attitudes and beliefs are more likely to advise patients to restrict physical and work activities and are therefore generally less adherent to clinical guidelines.^13,16,17^ Obviously, physiotherapy education has the potential to shift these misbeliefs toward evidence-based biopsychosocial attitudes and beliefs and subsequent biopsychosocial management of persistent pain. The proposed pain management competence profile to guide entry-level physiotherapy education is an important step to support this shift. For all of these reasons, we call for a worldwide implementation of the proposed pain management competence profile to guide entry-level physiotherapy education.

The journal editorial board and the CPS Awards Committee offer their most sincere congratulations and thanks to Mr. Augeard, Dr. Wideman, and colleagues for their award-winning article and for the decision to publish their work in the *Canadian Journal of Pain*. We look forward to next year’s CPS meeting in Ottawa, Ontario, when we hope to present two awards: one for the basic/preclinical sciences and another for the applied/clinical sciences. Submit your original research to the *Canadian Journal of Pain* and you may be the next winner of the Ronald Melzack–*Canadian Journal of Pain* Paper of the Year Award/Prix Ronald Melzack Pour L’Annee 2022 Des Articles Parus Dans la *Revue Canadienne de la Douleur*.
